# Advances in Diagnostic Technologies of Major Avian Respiratory Viruses: A Review

**DOI:** 10.3390/vetsci13101023

**Published:** 2026-09-27

**Authors:** Zhiqiang Hu, Ase Hailai, Huiling He, Chaoyun Yang, Yiqun Chen, Zengwen Huang, Xinhong Li, Shangen Xu, Ran Guan

**Affiliations:** 1 College of Animal Science, Xichang University, Xichang 615013, China; 2 Key Laboratory of Animal Epidemic Disease Detection and Prevention in Panxi District, Xichang University, Xichang 615013, China; 3 Liangshan Ecological Breeding Engineering Technology Research Center, Xichang University, Xichang 615013, China; 4 Zhejiang Key Laboratory for Restoration of Damaged Coastal Ecosystems, School of Life Sciences, Taizhou University, Taizhou 318000, China; 5 Zhejiang International Science and Technology Cooperation Base for Biomass Resources Development and Utilization, School of Life Sciences, Taizhou University, Taizhou 318000, China; 6 Taizhou Key Laboratory of Biomedicine and Advanced Dosage Forms, School of Life Sciences, Taizhou University, Taizhou 318000, China

**Keywords:** avian respiratory viruses, diagnostic technology, laboratory diagnostic, point-of-care diagnostic technique

## Abstract

Avian respiratory diseases, particularly those induced by the avian influenza virus (AIV), Newcastle disease virus (NDV), infectious bronchitis virus (IBV), and infectious laryngotracheitis virus (ILTV), represent a substantial economic threat to the global poultry industry. Several of these viruses can also transmit across species, posing risks to public health security. Given the challenges posed by similar clinical symptoms from different pathogens, the prevalence of co-infections, and rapid viral mutations, rapid, precise, and highly sensitive diagnostic techniques are imperative for effective disease prevention and control, epidemic surveillance, and epidemiological research. This review provides an overview of molecular and immunological diagnostic techniques for major avian respiratory viruses. All covered technologies are evaluated and compared across key performance metrics: target type, analytical sensitivity, multiplex capacity, turnaround time, point-of-care diagnostic techniques (POCT) suitability and application scenarios. Specifically, the review details real-time quantitative PCR (qPCR) and its multiplex formats, isothermal amplification methods including loop-mediated isothermal amplification (LAMP), recombinase-aided amplification (RAA) and recombinase polymerase amplification (RPA), and innovative CRISPR-Cas-based nucleic acid detection systems. It also examines high-throughput sequencing, microarray technologies, and advanced immunological assays enhanced by nanomaterials, recombinant proteins, and single-domain antibodies. The review further discusses integrated, automated, and portable diagnostic platforms, including microfluidics, and smartphone-assisted systems. Finally, the review outlines future trends in avian diagnostic technologies, providing a practical reference for building more efficient and accessible avian disease surveillance and early warning systems.

## Simple Summary

Avian respiratory diseases caused by avian influenza virus (AIV), Newcastle disease virus (NDV), infectious bronchitis virus (IBV), and infectious laryngotracheitis virus (ILTV) are major threats to the global poultry industry and to public health. Because these viruses cause similar clinical signs, often occur as co-infections, and mutate rapidly, fast and accurate diagnosis is essential for disease control and surveillance. This review summarizes current advances in molecular and immunological diagnostic technologies for these four viruses, including real-time PCR and multiplex assays, isothermal amplification methods, CRISPR-based detection, high-throughput sequencing, and immunoassays using recombinant proteins, nanobodies, and immunochromatographic strips (ICS). We compare the strengths and weaknesses of these technologies and discuss future trends toward integrated, automated, and connected diagnostic systems. The review aims to help researchers, veterinarians, and policymakers select appropriate diagnostic tools for outbreak investigation, routine surveillance, vaccination monitoring, and export certification, and to support more efficient surveillance and early-warning systems.

## 1. Introduction

Avian respiratory diseases, attributed to a range of viral pathogens including avian influenza virus (AIV), Newcastle disease virus (NDV, also known as Avian orthoavulavirus 1), infectious bronchitis virus (IBV), and infectious laryngotracheitis virus (ILTV), represent a significant threat to the global poultry industry. These diseases not only result in heightened poultry mortality, diminished egg production, and impaired growth performance, leading to substantial economic losses [1,2,3], but also pose potential risks to global public health security. Notably, certain AIV subtypes (e.g., H5, H7, H9, and H10) can cross the species barrier and infect humans, with the potential to precipitate a pandemic, thus becoming a global public health concern [4,5,6].

In addressing these challenges, the advancement and implementation of rapid, sensitive, specific POCT are essential. Traditional diagnostic methods, such as virus isolation and culture, while regarded as the “gold standard” are inherently time-consuming, complex, and necessitate high-level biosafety laboratories, rendering them inadequate for rapid epidemic response [7,8]. Rapid and precise diagnosis is fundamental for the effective control of epidemic outbreaks, the execution of accurate epidemiological surveillance, and the assessment of vaccine efficacy [4,9]. In recent years, a variety of innovative diagnostic technologies have been developed, including microfluidic chip-based real-time quantitative PCR (qPCR), CRISPR gene editing technology and loop-mediated isothermal amplification (LAMP), which offer the potential for rapid, on-site detection of avian respiratory diseases [7,10,11,12].

Therefore, this work is presented as a narrative review that synthesizes and compares recent advances in molecular diagnostics, immunological detection, and emerging sensing technologies for the diagnosis of the four major avian respiratory viruses. Relevant studies were retrieved from PubMed and Web of Science using keyword combinations including “AIV”, “NDV”, “IBV”, “ILTV”, and “diagnosis”/”detection”. We focused primarily on research published between 2015 and 2026 reporting novel methodological advances, and noted that a formal PRISMA-based systematic review protocol was not applied.

In contrast to previously published reviews on avian respiratory virus diagnostics, this review distinguishes itself in two primary aspects. Firstly, it provides a unified comparison framework that assesses each technology along common dimensions, including target type, analytical sensitivity, multiplex capacity, turnaround time, POCT suitability and application scenarios. Secondly, it emphasizes recently emerged technologies (such as CRISPR-based detection, microfluidic platforms, nanobody-assisted immunoassays, and smartphone-enabled systems). This review delves into the principles, advantages, and limitations of each technology, while also outlining their developmental trajectories and potential applications in avian disease control, public health early warning systems, and future diagnostic domains. Consequently, it serves as a valuable reference for researchers and practitioners in related fields.

## 2. Major Avian Respiratory Viruses and Their Diagnostic Targets

### 2.1. AIV

AIV is classified within the *Orthomyxoviridae* family and is characterized as a segmented, single-stranded negative-sense RNA virus. The viral genome is composed of eight distinct RNA segments, each encoding a variety of structural and non-structural proteins. The subtyping of AIV is predominantly determined by two surface glycoproteins: hemagglutinin (HA) and neuraminidase (NA). Currently, 16 HA subtypes (H1–H16) and 9 NA subtypes (N1–N9) have been identified in avian species [13]. Diagnostic targets for AIV are categorized into conserved and variable targets, depending on the objectives of detection. In nucleic acid testing, the Matrix (M) gene is one of the most conserved targets and is widely used for universal detection of all influenza A viruses, serving as the “gold standard” for molecular diagnostics [14,15,16,17]. The nucleoprotein (NP) gene is also highly conserved and serves as a universal detection target, as exemplified by its use in CRISPR-Cas13a-based detection technologies [18]. To address the needs of field and resource-limited settings, LAMP assays targeting the M gene and lyophilized qRT-PCR reagents have been developed to improve detection convenience and stability [14,19]. Viral subtyping predominantly depends on the amplification and sequencing of the highly variable HA and NA genes. Several rapid detection techniques, including recombinase-aided amplification (RAA), recombinase polymerase amplification (RPA) and multiplex PCR, have been developed to facilitate the swift identification of significant subtypes such as H3, H5, H7, and H9 [20,21,22,23,24,25]. Universal primers and sequencing methods for the NA gene have also been established to support comprehensive viral genotyping [13].

For immunological detection, the conserved NP protein is an ideal universal target for AIV antibody detection, enabling broad-spectrum enzyme-linked immunosorbent assay (ELISA) kits [26,27]. As a major surface antigen, the HA protein is a critical target for subtype-specific antibody detection and hemagglutination inhibition (HI) assays, which are essential for assessing vaccine efficacy and serological tracing of subtype-specific infections [28,29,30].

### 2.2. NDV

NDV belongs to the *Paramyxoviridae* family and is a non-segmented, single-stranded negative-sense RNA virus. Its genome encodes six major structural proteins, including NP, phosphoprotein (P), matrix protein (M), fusion protein (F), hemagglutinin-neuraminidase protein (HN), and large polymerase protein (L). NDV is classified primarily by pathogenicity into velogenic, mesogenic, and lentogenic strains. The amino acid sequence at the cleavage site of the F protein is the key molecular basis determining its pathogenicity. For nucleic acid detection of NDV, the M and F genes are the two principal targets. Because the M gene is highly conserved across genotypes, it serves as a universal NDV detection target on multiple molecular platforms. The F gene, in turn, serves not only in virus detection but also in differentiating velogenic from lentogenic strains, owing to the specificity of its cleavage-site sequence [31]. The highly conserved L gene has also been used to develop field-based LAMP assays that avoid nucleic acid extraction, making them particularly suitable for resource-limited settings [32].

For immunological detection, the NP and HN proteins of NDV are the primary targets. The NP protein is highly abundant in viral particles and strongly immunogenic, making it an ideal antigen for ELISA kits that detect NDV antibodies [33]. The HN protein, a critical surface protein, mediates viral attachment and release and is the principal antigen that elicits neutralizing and hemagglutination-inhibiting antibodies. It is therefore widely used as a target in HI assays and virus neutralization tests to evaluate protective immune responses [34,35,36].

### 2.3. IBV

IBV is a member of the *Coronaviridae* family and is characterized as a non-segmented, single-stranded positive-sense RNA virus, possessing a large genome of approximately 27.6 kb. The genome of IBV encodes several structural proteins, notably the spike (S), membrane (IBV-M), envelope (E), and nucleocapsid (N) proteins. IBV is distinguished by its remarkable genetic diversity, with its classification largely reliant on the S protein gene, particularly the hypervariable region (HVR) sequence of the S1 subunit. This genetic variability has resulted in the emergence of numerous serotypes and genotypes [37]. The genetic plasticity of IBV is not confined to the virus itself but can also facilitate the emergence of novel viruses. For instance, turkey coronavirus (TCoV) is hypothesized to have evolved from IBV through recombination of the S gene, which enabled it to infect turkeys and shifted its tissue tropism from the respiratory tract to the intestinal tract [38]. In China, extensive surveillance of broiler flocks across eight provinces from 2020 to 2021 revealed that the predominant IBV genotypes include the QX type (GI-19), TW type (GI-7), and 4/91 type (GI-13), with the QX type being the most prevalent strain [39]. Surveillance in Bangladesh has likewise confirmed the concurrent circulation of multiple genotypes, including QX-like, 4/91-like, and Mass-like strains, mirroring the global distribution of these lineages [40]. In 2021, respiratory disease outbreaks caused by GI-19 lineage variants were reported in chicken flocks in southern China despite vaccination with the H120 vaccine, highlighting the challenge that continuous IBV evolution poses to vaccine efficacy [41].

The molecular diagnosis of IBV relies on both conserved and variable gene targets. To facilitate broad-spectrum detection of all IBV strains, highly conserved genes such as the N gene or IBV-M gene are typically selected as targets [39,41,42]. Detection methodologies based on these conserved genes have been extensively employed in conventional RT-PCR and various rapid isothermal amplification techniques [43]. However, because these conserved genes are highly homologous within the genus Avian coronavirus (over 90% homology with TCoV), methods targeting the N or IBV-M gene may cross-react with closely related viruses such as TCoV [38]. Genotyping of IBV should prioritize the S1 gene. The viral genotype can be accurately identified by PCR amplification and sequencing of the HVRs within the S1 gene, specifically HVR1–2 and HVR3. This genotypic information is crucial for guiding vaccine selection and administration [40,44,45]. For instance, sequencing of the S1 gene from isolates during a 2021 outbreak in Guangdong demonstrated only approximately 77% nucleotide homology with the widely used vaccine strain H120, thereby elucidating the underlying cause of vaccine failure [41]. Targeted analysis of the S1 gene is therefore essential for both IBV genotyping and differentiating IBV from TCoV infections [38]. A comprehensive survey of S1 gene sequences across multiple Chinese provinces identified a predominance of QX-type strains (58.15% of isolates) and a notable prevalence of TW-type strains (25%), providing a detailed depiction of regional viral epidemiology [39]. S1 gene sequence analysis is important not only for genotyping but also for identifying viral recombination events and critical amino acid mutations. The study in question identified gene recombination between prevalent QX-type and TW-type strains, resulting in the emergence of novel recombinant viruses. Pathogenicity assays indicated that this recombinant virus, exemplified by strain CK/CH/JS/YC10-3, exhibited pathogenicity levels comparable to its parental QX type, manifesting as severe kidney swelling and airsacculitis. This underscores the pivotal role of gene recombination in viral evolution and its potential to precipitate new outbreaks [39]. Recent investigations have further corroborated that recombination occurs not only between different genotypes but also frequently within the same lineage. For instance, the newly emerged GI-19 lineage strain HH09 has been confirmed as a recombinant product of two distinct strains within this lineage (CK/CH/SCDY2003-2 and I0305/19). Novel amino acid mutations, such as T273I, T292A, and S331K, have also been identified in the hypervariable region of its S1 protein. These molecular-level alterations collectively drive the ongoing evolution of the virus [41]. For example, an analysis of prevalent strains in Bangladesh revealed nucleotide sequence differences of up to 37–41% in the S1 gene’s hypervariable region among different genotypes. This analysis also identified genotype-specific mutations, such as the G65E mutation in Mass-like strains and the Y328S mutation in 4/91-like strains [40]. Examination of QX-like strains further revealed specific mutations, such as 294F and 306Y, associated with high pathogenicity and potentially affecting neutralizing epitopes. These mutations potentially enable the virus to evade immunity, thereby facilitating its continued spread in vaccinated flocks [40]. The utilization of whole-genome sequencing technology offers more comprehensive insights for analyzing viral recombination and conducting evolutionary studies [37]. Although the S1 gene remains the gold standard for genotyping, its high mutation rate complicates molecular epidemiological studies and may cause discrepancies between phylogenetic history and viral geographic distribution. To address this issue, recent studies have suggested utilizing the RNA-dependent RNA polymerase (RdRp) gene within the viral replicase complex as an alternative target for molecular characterization. Research demonstrates that focusing on a variable region flanked by highly conserved regions within the RdRp gene can effectively differentiate field epidemic strains from vaccine strains. This approach also yields phylogenetic results that correlate strongly with the geographic origin of the strains, providing a more stable genetic marker for virus tracing and transmission studies [46]. Consequently, integrating RdRp gene analysis with traditional S1 gene genotyping can offer a more comprehensive and reliable framework for the molecular epidemiological surveillance of IBV. Integrating RdRp gene analysis with traditional S1 gene genotyping thus offers a more comprehensive and reliable framework for molecular epidemiological surveillance of IBV.

For serological diagnosis, the highly conserved and immunogenic N protein is the preferred target for broad-spectrum ELISA assays that detect antibodies against various IBV genotypes [47,48]. The S protein, particularly specific peptides located on its relatively conserved S2 subunit, has also been demonstrated to be effective for the development of ELISA methods that can broadly detect antibodies against different IBV serotypes [49]. Certain non-structural proteins, such as nsp5, have also been used as coating antigens for antibody detection [26].

### 2.4. ILTV

ILTV, also referred to as Gallid herpesvirus 1 (GaHV-1), is classified within the genus *Iltovirus*, part of the subfamily *Alphaherpesvirinae* in the family *Herpesviridae*. It is characterized by a linear double-stranded DNA genome, approximately 150–155 kb [50]. Unlike other RNA respiratory viruses, ILTV possesses DNA as its genetic material, which is relatively stable, although genetic variations are present among different strains. The virus establishes latent infections in the trigeminal ganglion after infection, making recovered chickens long-term carriers and potential sources of infection [50].

Nucleic acid detection is a mainstream approach for ILTV laboratory diagnosis. Diagnostic efforts commonly target the thymidine kinase (TK) gene and various glycoprotein genes, including gB (UL27), gC (UL44), gD (US6), and gE (US8) [50,51,52,53]. For these genetic targets, a range of PCR, qPCR, and isothermal amplification techniques, such as LAMP and RAA, have been developed to facilitate rapid and sensitive detection of ILTV, even in field settings [54,55,56]. Detection of ILTV DNA alone does not necessarily indicate active infection, because vaccine strains and latently infected carriers can also yield positive DNA signals. Sequence analysis of specific genes, such as US5, can therefore distinguish wild-type from vaccine strains [57]. Techniques such as TaqMan SNP genotyping identify single-nucleotide polymorphisms across multiple genes (e.g., US5, US6, US7, US8), enabling analysis of viral recombination events [50].

For immunological detection, ILTV glycoproteins are the primary antigens that elicit host immune responses. The gG protein plays a dual role, mediating viral invasion and acting as a broad-spectrum viral chemokine-binding protein (vCKBP). This function lets gG modulate innate immune responses through interactions with host chemokines, offering new perspectives for innovative immunological diagnostic targets and improving our understanding of viral immune evasion [50]. The gD protein is also strongly immunogenic. ELISA assays based on recombinant gD protein or its specific peptides effectively detect ILTV-specific antibodies in serum. These assays are well suited for monitoring vaccine-induced immune responses and conducting epidemiological studies [58,59].

## 3. Traditional Diagnostic Technologies

Traditional diagnostic methods constitute the cornerstone for diagnosing viral respiratory diseases in poultry, forming the basis for etiological confirmation, epidemiological surveillance, and the assessment of vaccine efficacy. However, these classical approaches exhibit significant limitations concerning turnaround time, sensitivity, specificity, and applicability in field conditions.

### 3.1. Virus Isolation and Identification

Virus isolation and culture are widely regarded as the “gold standard” for etiological diagnosis. This method is also the officially recognized confirmatory standard for AIV, NDV, IBV, and ILTV as outlined in the Manual of Diagnostic Tests and Vaccines for Terrestrial Animals by the World Organisation for Animal Health (WOAH). It thus serves as the definitive basis for international trade quarantine and statutory outbreak confirmation. Virus inoculation into specific-pathogen-free (SPF) embryonated chicken eggs is considered the most classical and reliable technique [60,61]. This approach yields infectious live virus, providing essential material for subsequent virus identification, pathogenicity testing, antigenic analysis, and whole-genome sequencing. Despite its high reliability, the inherent limitations of virus isolation constrain its application in contemporary rapid disease prevention and control efforts. Firstly, the method is time-intensive: the entire process, from sample inoculation to virus propagation, harvest, and identification, typically spans several days or even weeks, rendering it inadequate for the demands of early warning and rapid diagnosis [60,62,63,64]. Secondly, and most critically, the isolation of highly virulent pathogens, such as the highly pathogenic AIV (HPAIV), necessitates execution in biosafety level 3 laboratories, significantly limiting its feasibility in grassroots laboratory settings [60]. Although virus isolation remains the authoritative standard for definitive confirmation, its deficiencies in timeliness and accessibility have prompted both academia and industry to develop more rapid diagnostic technologies, with virus isolation serving as the benchmark for evaluating their performance [65].

### 3.2. Traditional Serological Detection Methods

Serological assays evaluate the immune status of the host by identifying specific antibodies present in serum. These assays are crucial for disease surveillance, assessing vaccine efficacy, and conducting retrospective epidemiological investigations. Traditional serological techniques primarily encompass the HI test and the virus neutralization (VN) test. The HI test is simple and cost-effective and is widely used to detect antibodies against viruses with hemagglutinating activity, such as AIV and NDV [66,67]. The VN test, which quantifies the capacity of serum to neutralize viral infectivity, is considered the “gold standard” for evaluating protective antibody levels, as its outcomes directly correlate with the host’s resistance to viral challenges [68,69]. Both the HI and VN tests are serological methods officially endorsed by the WOAH for monitoring population immunity in poultry and formally assessing vaccine efficacy. Traditional serological tests are subject to several limitations. Firstly, a “window period” for antibody production occurs, during which the host has not yet produced sufficient antibodies, thereby rendering serological tests ineffective for early diagnosis. Secondly, the VN test is both labor-intensive and time-consuming, requiring stringent experimental conditions such as specific cell lines or embryonated eggs, which hinders high-throughput detection [68]. Thirdly, the HI test does not consistently provide an accurate measure of neutralizing protective antibody levels [69]. The most significant challenge, however, lies in the limited ability to differentiate infected from vaccinated animals (DIVA). When traditional inactivated vaccines that are antigenically similar to field strains are employed, these methods fail to effectively distinguish between antibodies derived from infection and those from vaccination, complicating epidemiological analysis. For viruses such as IBV, which comprise multiple serotypes with limited cross-protection, traditional serological tests show restricted type specificity and cannot identify the circulating strain, thus affecting vaccine selection and application [41].

### 3.3. Traditional RT-PCR and ELISA Methods

To address the limitations associated with traditional diagnostic methods, reverse transcription PCR (RT-PCR) and ELISA have been established as standard laboratory diagnostic tools. RT-PCR facilitates the direct, rapid, and highly specific detection of pathogens by selectively amplifying viral nucleic acid fragments, offering a significantly faster alternative to virus isolation [70]. Nevertheless, conventional RT-PCR requires precise instrumentation, a reliable power supply, and skilled personnel, restricting its use mainly to centralized laboratories and limiting its application for POCT in field or resource-limited settings [63]. Conventional gel-electrophoresis RT-PCR is also generally less sensitive and precise than RT-qPCR [71].

Traditional ELISA techniques typically utilize polyclonal antibodies or whole-virus lysates, which can lead to cross-reactivity among various subtypes or viral strains, thereby undermining specificity [72,73]. The sensitivity of these methods for antigen detection is also generally inferior to that of molecular techniques, requiring relatively high concentrations of viral protein in the sample for accurate detection [74,75]. Although ELISA offers a quicker alternative to HI and VN tests, it still requires laboratory-based execution. This limitation has spurred the development of rapid detection technologies more suitable for field applications, such as immunochromatographic test strips that rely on antigen–antibody interactions [75,76].

## 4. Molecular Diagnostic Technology

### 4.1. The qPCR and Its Multiplex Applications

The qPCR enables precise nucleic acid quantification via real-time fluorescence monitoring and is widely established as a reference method for quantitative molecular diagnosis of avian diseases [16]. It enables the measurement of viral load, assessment of viral replication, and evaluation of vaccine efficacy [77,78], and is applicable to a wide range of sample types, including routine animal samples as well as environmental samples such as poultry house dust [79]. Notably, diagnostic performance varies across sample matrices due to differences in viral load and background inhibitors, with each sample type suited to specific diagnostic scenarios. From a deployment perspective, qPCR assays are primarily applied in laboratory settings, as they rely on precision thermal cyclers and calibrated fluorescence detection systems. While portable qPCR platforms have emerged for field applications, routine high-throughput and highly accurate quantitative testing, with a typical turnaround time of 1–2 h, remains predominantly laboratory-based. Currently, qPCR methodologies are predominantly employed for broad-spectrum pathogen detection and virological research (Table 1). To address challenges posed by viral mutations, Alexander Morris et al. enhanced detection sensitivity for NDV from 93% to 100% by optimizing primers and probes [80], and developed a RT-qPCR assay capable of differentiating between high and low pathogenicity H7 AIV without the need for sequencing [81]. Wang et al. utilized strand-specific RT-qPCR to elucidate the replication strategy of AIV within the host [6]. A low-cost SYBR Green I RT-PCR for IBV, suitable for resource-limited areas, has been developed, with sensitivity 10 times higher than traditional methods [71].

Multiplex qPCR facilitates the concurrent detection of multiple pathogens within a single reaction tube, thereby addressing the complexities of co-infections and supporting epidemiological research in large-scale agricultural settings (Table 1). This method has been effectively employed in extensive outbreak investigations [82], with research indicating that up to 94% of affected chickens exhibit co-infections involving three or more pathogens [83]. Currently, available multiplex systems include the detection of AIV, NDV, and duck Tembusu virus (DTMUV) [84]; ILTV, NDV, and avian metapneumovirus (aMPV) [85]; H9 AIV and IBV [86]; ILTV, fowlpox virus (FWPV), reticuloendotheliosis virus (REV), and REV-integrated FWPV [87]; and for goose parvovirus (GPV), H5 AIV, and goose astrovirus (GoAstV) [21]. Additionally, multiplex RT-PCR can differentiate between various genotypes of the same pathogen, exemplified by a pentaplex RT-PCR that distinguishes IBV field strains from vaccine strains [44], or when combined with high-resolution melting (HRM) technology for rapid genotyping [88]. Specific assays for the emerging IBV genotype GVIII have also been developed [89]. In the context of AIV surveillance, multiplex RT-qPCR assays have been designed to differentiate between H5/H7/H9 [90], H4/H6/H10 [91], H3/H4/H5 [24] and region-specific prevalent strains [92].

**Table 1:** Diagnostic Methods Based on qPCR and dPCR Technologies.

Method	Targets	Limit of Detection (LOD)	Reference
strand-specific RT-qPCR	vRNA, cRNA and mRNA of AIV-H7N9-PB2 and NA genes	PB2: 46.254 copies/µL for vRNA, 46.081 copies/µL for cRNA, 46.103 copies/µL for mRNA; NA: 45.748 copies/µL for vRNA, 46.782 copies/µL for cRNA, and 46.119 copies/µL for mRNA	[6]
SYBR Green I RT-PCR	IBV-N	100 copies/μL	[71]
Pathotyping RT-qPCR	H7-HA Cleavage Site	Sensitivity comparable to universal M gene qPCR	[81]
Single-plex RT-qPCR	IBV-S1 (GVIII subtype)	10 copies/μL	[89]
Single-plex RT-qPCR	AIV-H9-HA (epidemic strain in South Korea)	7.3–478.5 copies/μL	[92]
Duplex RT-qPCR	AIV-H9-HA and IBV-N	H9 AIV: 1.0 × 10^1^ EID_50_/mL; IBV: 1.5 × 10^1^ EID_50_/mL	[86]
Triplex RT-qPCR	AIV-M, NDV-F and DTMUV-E	10 copies/μL	[84]
Triplex RT-qPCR	GPV-VP1, AIV-H5-HA, GoAstV-ORF2	GPV/GoAstV: 10 copies/μL; H5 AIV: 1 copy/μL	[21]
Triplex RT-qPCR	AIV-H5/H7/H9-HA	50 copies/reaction	[90]
Triplex RT-qPCR	AIV-H3/H4/H5-HA	2.1 × 10^2^ copies/μL	[24]
Multiplex PCR	ILTV-US4, FWPV-NPH-I, REV-integrated FWPV-gag, REV-gp90	1 × 5^4^ copies/tube	[87]
Multiplex RT-qPCR	NDV-F (genotypes of VII.1.1, VII.2 and XIV)	genotype VII.2: 10^2.4^ EID_50_/mL; genotype VII.1.1:10^2.1^ EID_50_/mL; genotype XII: 10^2.5^ EID_50_/mL	[80]
Multiplex RT-qPCR	ILTV-gC, NDV-M, NDV-L (class I NDV) and aMPV (A and B)-N	10 copies/μL	[85]
Multiplex RT-qPCR	AIV-M, AIV-H4/H6/H10-HA	M: 1 copy/reaction H4/H6/H10: 10 copies/reaction	[91]
Multiplex RT-qPCR	IBV-S1 (genotypes of QX, G VI-1, Mass, 4/91 and LDT3)	10^3^ copies/μL	[44]
dPCR	IBV genome	0.55 copy/µL	[93]
Quadruplex ddPCR	AIV-H9-HA, IBV-M, *P. aeruginosa*-Pal, *E. coli* -UidA	AIV: 3.02 copies/μL; IBV: 3.08 copies/μL; *P. aeruginosa*: 3.19 copies/μL; *E. coli*: 3.39 copies/μL	[1]

Note: LOD values are presented as originally reported in the cited studies. Units differ across assays due to variable experimental conditions and are not directly comparable.

Digital PCR (dPCR) has been developed and implemented as a complementary molecular diagnostic platform to qPCR methods, with a typical detection workflow completed within 1–2 h, and is predominantly applied in laboratory testing (Table 1). In terms of quantitative capability, dPCR enables absolute quantification of target nucleic acids without the need for a standard curve, which is its core technical advantage. For analytical sensitivity, dPCR assays generally achieve lower LODs than conventional qPCR in well-controlled laboratory settings. For example, a dPCR assay established for IBV detection obtained a LOD as low as 0.55 copies/µL, which was markedly lower than that of the matched conventional qPCR assay under identical experimental conditions [93]. A quadruplex droplet digital PCR (ddPCR) assay has been designed to simultaneously and absolutely quantify four respiratory pathogens: H9 AIV, IBV, *Pseudomonas aeruginosa*, and *Escherichia coli* [1].

### 4.2. Isothermal Amplification Technology for POCT

While conventional qPCR is traditionally dependent on laboratory-based thermal cyclers, portable qPCR systems have recently become available and can extend quantitative detection to field settings. In contrast, isothermal amplification technology, which does not require a thermal cycler and typically completes the detection workflow within 30–60 min, is particularly well-suited for the development of POCT methods (Table 2). LAMP facilitates rapid amplification at temperatures between 60–65 °C, with results that can be visually interpreted. The reverse transcription LAMP (RT-LAMP) assay developed by Songzi Zhang et al. for AIV is ten times more sensitive than conventional RT-PCR [94]. Furthermore, LAMP, when optimized with appropriate primer design, exhibits sensitivity comparable to that of official real-time RT-PCR [17]. LAMP results are primarily interpreted visually, and colorimetric LAMP can visually detect as few as 490 copies/μL [14]. Additionally, the integration of LAMP with lateral flow dipstick (LFD) technology facilitates the simultaneous visual detection of IBV and NDV, achieving a detection threshold LOD of 10^0.7^–10^0.8^ copies per reaction [95]. Moreover, the combination of LAMP with gold nanoparticle (AuNP)-based DNA nanoprobe technology permits the on-site detection of ILTV with a minimum detection limit of 200 copies/reaction, allowing for the direct analysis of raw swab samples without the need for nucleic acid extraction, thereby significantly simplifying on-site procedures [54].

RPA and RAA are two representative commercialized isothermal amplification technologies based on the recombinase-mediated strand displacement principle. Both systems can complete nucleic acid amplification within 15–40 min at a constant temperature ranging from 37 to 42 °C, but they are developed as independent technical platforms with distinct reagent formulations and commercial systems. For the RAA system, RAA combined with LFD assays have been established for detection of H7 AIV [96], H9N2 AIV [97], ILTV [55] and IBV [43], demonstrating significantly higher sensitivity than conventional PCR methods (Table 2). The real-time fluorescence RAA (RF-RAA) exhibits even greater sensitivity and has been employed for the ultrasensitive and rapid detection of ILTV [51], NDV [98], IBV [42], H3 AIV [20] and H9 AIV [23] (Table 2). The dual-mode RT-RAA platform offers the flexibility to detect H5, H7, and H9 subtypes [22], and can be integrated with microfluidic chips to create a “lab-on-a-chip” system [99]. Li et al. devised a technique utilizing RT-RAA in conjunction with LFD for the detection of the H5 subtype AIV. This method facilitates the completion of the reaction within 15 min at 37 °C, allowing for visual assessment of results via test strips. It boasts a detection limit as low as 1 copy/μL, exhibiting a sensitivity 100 times greater than that of traditional RT-PCR, thereby rendering it exceptionally suitable for rapid screening in grassroots farming environments [63]. For the RPA system, researchers have also coupled this technology with straightforward visual readout methods (Table 2). For instance, Mohamed et al. integrated RPA technology with nucleic acid lateral flow immunochromatographic test strips (NALF) to develop a rapid on-site detection method. This approach amplifies the highly conserved NDV-M gene or IBV-N under constant temperature conditions at 38 °C, completing the entire amplification process within 20–40 min and achieving a detection limit as low as 10 copies/reaction [100]. A recognized limitation of isothermal amplification methods is the large quantity of amplification products generated, which raises the risk of carry-over contamination and false-positive results, particularly in low-resource field settings. This risk can be mitigated through closed-cartridge and integrated sample-to-answer platforms, physical separation of pre- and post-amplification workflows, uracil-DNA glycosylase (UDG)-based carryover control, and strict procedural segregation.

**Table 2:** POCT Methods Based on Isothermal Amplification Technology.

Method	Target	LOD	Reference
RT-LAMP	AIV-M and AIV-H5/H9-HA	100–1000 copies/reaction	[94]
RT-LAMP	AIV-M	10^0.8^ EID_50_/reaction	[17]
Real-time RT-LAMP/colorimetric-LAMP	AIV-M	RT-LAMP: 49 copies/μL; colorimetric LAMP: 490 copies/μL	[14]
Multiplex RT-LAMP-LFD	IBV-5′-untranslated region (UTR), NDV-large polymerase gene (LP)	10^0.7^–10^0.8^ copies/reaction	[95]
AuNP-based LAMP	ILTV-gE	200 copies/reaction	[54]
RT-RAA-LFD	AIV-H7-HA	10 copies/μL	[96]
RT-RPA-LFD	AIV-H9N2-HA	0.15 pg of H9N2 AIV RNA/reaction	[97]
RAA-LFD	ILTV- thymidine kinase (TK)	100 copies/μL	[55]
Real-time RT-RAA	ILTV-TK	10 copies/μL	[51]
RT-RAA-LFD	IBV-S2	100 copies/μL	[43]
Real-time RT-RAA	NDV-M	6.89 EID_50_/mL	[98]
Real-time RT-RAA	IBV-5′-UTR	2 copies/μL	[42]
Real-time/visualizable RT-RAA	AIV-H3-HA	Fluorescence: 224 copies/reaction; Visualization: 1527 copies/reaction	[20]
Real-time/visualizable RT-RAA	AIV-H9-HA	Fluorescence: 163 copies/reaction; Visualization: 1759 copies/reaction	[23]
RT-RAA-LFD/Real-time RT-RAA	AIV-H5/H7/H9-HA	LFD: 100 copies/μL; Fluorescence: 10 copies/μL	[22]
RPA-NALF	NDV-M and IBV-N	10 copies/reaction	[100]
RT-RAA-LFD	HA	1 copy/μL	[63]

Note: LOD values are presented as originally reported in the cited studies. Units differ across assays due to variable experimental conditions and are not directly comparable.

### 4.3. CRISPR-Based Diagnostic Systems

CRISPR-based diagnostic technology can be effectively integrated with isothermal amplification techniques. This approach employs Cas proteins to specifically identify amplification products, subsequently activating their trans-cleavage activity to degrade reporter probes and release detection signals. The technology is characterized by its high specificity and sensitivity, coupled with straightforward reaction conditions and rapid detection capabilities (30–60 min), rendering it highly suitable for POCT applications (Table 3). Recently, a detection platform for AIV based on RT-RAA-CRISPR-Cas13a has been developed, achieving a detection limit as low as 1 copy/μL using the fluorescence method and 10 copies/μL using the LFD method [3]. For the H5 [62] and NP [18] genes of AIV, the sensitivity of the RAA-CRISPR-Cas13a-LFD method reaches as low as 0.1 copy/μL and 1 copy/μL, respectively, which is significantly lower than that of conventional PCR methods. The “one-pot” method further simplifies the operational process and has been employed to detect H5 AIV, specifically identifying the prevalent 2.3.4.4b evolutionary branch [11] (preprint, not peer-reviewed). Overall, CRISPR-Cas13a platforms for AIV couple RT-RAA pre-amplification with Cas13a collateral RNA cleavage and provide both fluorescence and lateral-flow readouts for the conserved M/NP genes and the H5, H7 and H9 HA genes, combining single-base specificity with isothermal speed and thus representing a promising field-deployable route for avian influenza diagnosis.

### 4.4. High-Throughput and Sequencing Technologies

High-throughput technologies facilitate the simultaneous analysis of numerous samples, encompassing primarily microarray methods, Luminex liquid suspension chips, and the GeXP platform (Table 4). These technologies are predominantly laboratory-based, with a typical detection workflow completed within 4–6 h, and primarily used for parallel detection of multiple pathogens and subtypes in batch samples as well as large-scale epidemiological screening. Oligonucleotide microarrays are capable of concurrently detecting AIV, NDV, IBV, and their respective subtypes, exhibiting high sensitivity with results discernible to the naked eye [101,102]. The Luminex liquid suspension chip can detect up to four respiratory pathogens [103] simultaneously, with sensitivity and specificity comparable to single-plex qPCR. The Luminex xMAP technology is applicable for the detection of antibodies against multiple pathogens [104]. The GeXP platform, multiplex RT-PCR using a GeXP analyser, supports high-throughput detection of various AIV-HA [105] and -NA subtypes [106,107], achieving a sensitivity threshold of 100 copies/μL.

Next-generation sequencing (NGS) and nanopore sequencing are revolutionizing pathogen research [108]. These technologies are primarily applied in laboratory settings and is primarily used for the identification of unknown pathogens, whole-genome analysis of viruses, molecular epidemiological tracing and rapid pathogen sequencing and typing during acute outbreaks. Metagenomic NGS (mNGS), which is non-targeted, enables the identification of unknown pathogens. To address the challenge of host nucleic acid interference, targeted enrichment sequencing is employed to detect multiple AIV subtypes from environmental samples [109]. Nanopore sequencing offers portability and real-time analysis, with non-targeted pathogen identification capabilities comparable to those of the Illumina MiSeq platform. It is capable of detecting and genotyping NDV within one hour [110]. Nanopore sequencing can also be integrated with targeted amplification techniques to facilitate the efficient monitoring of specific viral families [111].

## 5. Immunological Detection Technology

### 5.1. Establishment and Application of ELISA Methods

Competitive or blocking ELISA is a technique utilized for the detection of specific antibodies by inhibiting the binding of enzyme-labeled monoclonal antibodies, thereby minimizing non-specific interference. This method, predominantly laboratory-based with a total turnaround time of 2–4 h, is widely employed for the detection of antibodies or antigens related to avian respiratory pathogens (Table 5). Henriques et al. developed a comprehensive AIV antibody detection approach based on conserved AIV proteins, achieving a sensitivity rate of 100% [112]. Epitope-blocking ELISA for the H5 subtype of AIV [29] and competitive ELISA for the H7 subtype of AIV [30] have been developed, both demonstrating high sensitivity. The blocking ELISA method, which utilizes antigens derived from NDV vaccines, is applicable for the in vitro potency evaluation of NDV vaccines, with results showing a strong correlation with the HA test [113]. Double-antibody sandwich ELISA is capable of directly detecting viral antigens, such as the HA protein of H9N2 [75,114], H3 subtype [115], and H6 subtype [116], exhibiting high sensitivity and specificity. To address the limitations inherent in traditional ELISA techniques, innovative methodologies utilizing novel antigens and antibodies are being developed. These advancements predominantly involve the use of recombinant proteins, synthetic peptides, nanobodies, and novel detection platforms.

(1) Application of recombinant proteins and synthetic peptides: Recombinant proteins offer a solution to the issues of batch-to-batch variability and biosafety concerns associated with whole virus antigens (Table 5). For instance, indirect ELISAs utilizing *E. coli*-expressed recombinant IBV-N protein [119] and the conserved IBV-nsp5 have demonstrated exceptional diagnostic efficacy [120]. The nsp5 antigen has further been employed in the construction of a protein microarray chip, leading to the development of a highly sensitive chemiluminescence immunoassay and a rapid diagnostic test, with the diagnostic sensitivity of 98.59% [122]. To mitigate the risk of false positives, the baculovirus expression system has been utilized to produce highly specific IBV-N protein [48]. Monoclonal antibodies targeting novel epitopes of conserved internal AIV proteins, such as PB1, provide essential materials for the development of universal detection reagents [123]. The methods for antigen immobilization play a pivotal role in preserving the natural conformation of antigens, particularly using concanavalin A (ConA), which facilitates the effective screening of functional monoclonal antibodies [34]. While synthetic peptides serve as safe alternatives to traditional antigens, their design must account for viral evolution, as mutations at critical sites, such as the 16K→16R mutation in the IBV-S2 protein, can significantly impact detection range [124]. A peptide-based enzyme-linked immunosorbent assay (pELISA) targeting the conserved S2 region has demonstrated broad detection capabilities across multiple IBV genotypes and enables early detection [118]. The implementation of multi-antigen peptide (MAP) strategies [117] and the optimization of novel peptides [49] can further enhance assay performance. A pELISA utilizing broad-spectrum neutralizing epitope peptides has shown a strong correlation with neutralizing antibody titers (r = 0.83), thereby facilitating the evaluation of vaccine protection efficacy [68]. To more accurately measure protective neutralizing antibodies, an NDV neutralization test employing an eGFP reporter virus (eGFP-NT) has been developed, exhibiting a high correlation with traditional neutralization tests (R^2^ = 0.994) and significantly outperforming ELISA and HI tests [69]. Moreover, recombinant monoclonal antibodies, such as anti-H7 AIV antibodies, are increasingly supplanting animal-derived antibodies to ensure consistency across reagent batches and to adhere to animal welfare standards [28].
(2) Application of nanobodies (Table 5): Nanobodies are considered ideal tools due to their diminutive molecular size and high stability [125]. The fusion of nanobodies with horseradish peroxidase (HRP) facilitates one-step detection, thereby significantly streamlining the process and enhancing sensitivity. One-step competitive enzyme-linked immunosorbent assays (cELISAs) targeting NDV-NP protein [121] and IBV-N protein [47] reduce detection time to one hour and offer significant advantages in the early detection of infections.
(3) Novel detection platforms (Table 5): A pioneering fenobody (multivalent capture)-RANbody (reporter molecule fusion) dual-nanobody sandwich ELISA platform markedly enhances NDV detection sensitivity [72]. Digital ELISA further elevates sensitivity, enabling the detection of single-virus particles of H7N9 AIV at the aM level [12]. Liquid-phase chip technology based on Luminex xMAP facilitates high-throughput parallel detection of antibodies against multiple pathogens, such as ILTV and IBV, within a single well, achieving sensitivity 2–4 times greater than that of traditional ELISA [59].

### 5.2. Application of Immunochromatographic Strip (ICS) for On-Site Rapid Detection

The ICS is pivotal in POCT, with its efficacy significantly improved through the optimization of antibodies and materials. The ICS designed for AIV H7N9 strain achieves detection within 10 min, with a detection threshold of 2.4 lgEID_50_/0.1 mL and exhibits high specificity [76]. In the case of H9N2 AIV, the ICS demonstrated diagnostic sensitivity and specificity of 90.9% and 97.4%, respectively, when compared to qRT-PCR in clinical samples [75]. The ICS for IBV detection employs a combination of N and S protein monoclonal antibodies as capture antibodies, facilitating broad-spectrum detection across multiple genotypes and enabling virus detection as early as one day post-infection [126]. For NDV, the ICS is capable of detecting antigens 36 h post-infection, prior to the onset of symptoms, underscoring its utility for early diagnosis [127].

### 5.3. Integrated, Automated, and Intelligent Diagnostic Platforms

Conventional etiological diagnostic techniques, such as virus isolation and serological testing, depend on centralized laboratory facilities, costly equipment, and prolonged turnaround times, making them inadequate for on-site rapid diagnosis and extensive surveillance. To overcome these limitations, novel integrated, automated, and intelligent diagnostic platforms have been developed. These platforms consolidate various stages, including sample processing, biological reactions, and signal detection, to achieve a fully automated “Sample-in, Result-out” process. This advancement offers robust technical support for the prevention and control of avian diseases.

#### 5.3.1. Lab-on-a-Chip

Microfluidic technology, commonly referred to as lab-on-a-chip (LOC), effectively integrates sample preparation, reaction, and detection processes onto a microchip, thereby facilitating automation and miniaturization in the diagnosis of avian diseases. During the sample pretreatment phase, MiVET technology is capable of concentrating the AIV by a factor of 100 to 1000, thereby establishing a basis for high-sensitivity detection [128]. In the realm of nucleic acid detection, several integrated platforms have been developed to enable rapid on-site diagnosis. The FIGHTER platform incorporates RT-qPCR, allowing for the quantitative detection of H9N2 AIV within 30 min [10]. The Alveo Sense platform employs RT-LAMP alongside electrical impedance sensing to detect AIV (M, H5, H7, H9) in untreated swabs within 45 min [15]. A LAMP microfluidic chip designed for ILTV achieves high-sensitivity detection within 30 min [129]. Furthermore, an RPA-integrated microarray chip is capable of distinguishing H5, H7, and H10 AIV subtypes with high sensitivity within 10 min [99]. To tackle the challenge of complex co-infections, a microfluidic high-throughput PCR platform has been developed, capable of concurrently detecting 96 pathogens across 96 samples [130]. The RITA-2 low-density RT-PCR array also facilitates comprehensive subtyping of AIV, covering 16 HA and 9 NA subtypes, while also enabling the simultaneous detection of NDV and IBV [131]. This technology is further employed in immunological analyses, exemplified by the use of protein microarrays to differentiate between AIV H5 and H7 antibodies [132]. Moreover, the integration of droplet PCR with encoded microbeads substantially augments the throughput of multiplex detection [133].

#### 5.3.2. Integrated Application of Smartphones

Smartphones equipped with detection modules function as controllers, analyzers, and communication gateways, thereby enhancing the capabilities for objective quantification and data management in on-site testing. For result interpretation, a lateral flow technology utilizing enhanced upconversion nanoparticles, in conjunction with a mobile phone application, can quantitatively detect H5/H7/H10 AIV with high sensitivity within 10 min, achieving a detection limit as low as 0.0156 ng/mL. The validation results with clinical samples are entirely consistent with those obtained from RT-PCR [4]. In terms of system control and data management, a portable LAMP system connects to a smartphone via Bluetooth, facilitating on-site detection of pathogens such as H5N1 with sensitivity comparable to qPCR. This system enables an integrated “detection-analysis-localization-upload” process: once detection results are linked with GPS information, they are uploaded to the cloud in real time, thereby constructing a dynamic epidemic map for real-time monitoring and early warning [7].

## 6. Conclusions

In summary, viewed horizontally with the above technologies (Table 6), the platforms are complementary rather than interchangeable. Nucleic-acid amplification (RT-PCR/RT-qPCR and dPCR) offers the highest analytical sensitivity and quantification in centralized laboratories; isothermal LAMP/RPA/RAA and CRISPR-based assays trade some multiplexing throughput for rapid, instrument-light field detection; high-throughput microarray/xMAP/GeXP and sequencing maximize multiplexing and subtyping/resolution at the cost of equipment and turnaround; ELISA and immunochromatographic strips provide low-cost antigen/antibody testing, with strips optimized for rapid screening at somewhat lower sensitivity; and integrated microfluidic, and smartphone platforms aim to close the sample-to-answer gap.

Different diagnostic scenarios impose distinct requirements for sensitivity, throughput, turnaround time and portability. For outbreak investigation in field settings, rapid, portable and easy-to-operate technologies such as RAA-LFD, RT-LAMP and ICS are preferred, enabling preliminary pathogen identification within 30–60 min without sophisticated equipment. For routine flock surveillance in laboratory settings, multiplex RT-qPCR is the mainstream choice, supporting simultaneous detection of multiple pathogens with high throughput and quantitative capability. For vaccination efficacy monitoring, both quantitative molecular assays (for viral load assessment) and serological methods (for antibody level detection) are widely used. Furthermore, the molecular and serological diagnostic methods described above form the technical basis for DIVA strategies, supporting the differentiation of field viral infection from vaccine-induced immunity in poultry disease surveillance and vaccination efficacy evaluation. For export certification and pathogen confirmation, virus isolation, Sanger sequencing or NGS are typically applied as confirmatory methods, providing definitive genotypic or biological evidence.

## 7. Prospects and Perspectives for Future Research

Despite notable advancements in diagnostic technologies for avian respiratory viruses, the increasingly complex disease landscape and global challenges necessitates future efforts to prioritize breakthroughs in specific areas.

(1) The development of an Integrated Surveillance Network under the “One Health” concept

The cross-species transmission of viruses, such as AIV, presents a substantial threat to public health [134]. Therefore, future pathogen surveillance should extend beyond farmed animals to establish a comprehensive, multi-dimensional surveillance network that includes animals, humans, and the environment. For instance, employing metagenomics or targeted sequencing on environmental samples, such as wetland sediments, can proactively identify and alert us to potential pathogens carried by wild birds. This approach provides a scientific foundation for interrupting virus transmission chains at their source [109].

(2) The Utilization of Artificial Intelligence and Big Data in Epidemic Early Warning Systems:

As high-throughput sequencing and large-scale epidemiological survey data continue to accumulate [135], the effective utilization of this extensive information presents a novel challenge. Future advancements in artificial intelligence (AI) and big data analytics hold the potential to integrate multi-dimensional data sets, encompassing viral genetic sequences, geographical data, host characteristics, and climatic conditions. Such integration will facilitate the development of precise models for virus transmission and evolution, thereby enabling the early warning of outbreak risks and the dynamic prediction of transmission pathways. Beyond epidemiological forecasting, AI-based image and signal recognition is increasingly used to interpret strip, fluorescence and sensor readouts objectively, reducing subjective judgment at the point of need.

(3) Development of Innovative Diagnostic Reagents to Combat Rapid Viral Mutations:

RNA viruses, such as IBV and AIV, are characterized by high mutation rates. The continuous emergence of novel variants may compromise the efficacy of existing diagnostic primers and probes, potentially resulting in undetected cases [136]. A primary objective of future research and development is to create broad-spectrum or rapidly adaptable diagnostic reagents. Bioinformatics approaches, including single nucleotide polymorphism (SNP) analysis, can be employed to identify highly conserved regions within viral genomes as potential targets [137]. Simultaneously, technologies like CRISPR provide significant flexibility for the swift updating of diagnostic reagents to address emerging variants, due to the simplicity of redesigning and synthesizing guide RNAs.

(4) Promoting the Global Dissemination of High-Sensitivity, Low-Cost, and User-Friendly Diagnostic Technologies

Effective global disease control requires transforming advanced diagnostic technologies into widely accessible tools, particularly in resource-limited settings. Future technological advancements must not only aim for high sensitivity and specificity but also emphasize affordability, operational simplicity, and minimal environmental demands. The promotion of POCT platforms, particularly those based on smartphones and portable devices, will be instrumental in achieving this objective. This approach holds significant potential for enhancing global animal and public health security.

(5) Overcoming laboratory-to-field translation bottlenecks and advancing integrated platforms

A key bottleneck is integrating robust sample preparation, ambient-temperature-stable reagents, quality-controlled manufacturing and regulatory/field validation into a single workflow. Future systems will increasingly converge nucleic-acid amplification, CRISPR or biosensor recognition, and automated readout within one closed platform, minimizing manual handling and contamination while maximizing field deployability.

## Figures and Tables

**Table 3:** Diagnostic Methods Based on CRISPR Technology.

Method	Target	LOD	Reference
RT-RAA-CRISPR-Cas13a	AIV-M, AIV-H5/H7/H9-HA	Fluorescence: 1 copy/μL; LFD: 10 copies/μL	[3]
RAA-CRISPR-Cas13a-LFD	AIV-H5-HA	0.1 copy/μL	[62]
RAA-CRISPR-Cas13a-LFD	AIV-NP	1 copy/μL	[18]
“One-pot” method	AIV-H5-HA (2.3.4.4b evolutionary branch)	121.7 copies/μL	[11]

**Table 4:** Diagnostic Methods Based on High-Throughput and Sequencing Technologies.

Method	Target	LOD	Reference
Oligonucleotide microarrays	AIV-M, AIV-H5/H7/H9-HA, NDV-F, IBV-N	AIV/NDV: 0.1 EID_50_; IBV: 1 EID_50_	[101]
Oligonucleotide microarrays	RNAs of H5N1, H7N9, H9N2	H5N1: 1 × 10^−8^ ng/μL for H5, 1 × 10^−7^ ng/μL for N1; H7N9: 1 × 10^−8^ ng/μL for H7 and 1 × 10^−7^ ng/μL for N9; H9N2: 1 × 10^−8^ ng/μL for H9 and 1 × 10^−8^ ng/μL for N2	[102]
Luminex liquid suspension chip	AIV-M, NDV-M, IBV-5′-UTR, ILTV-ICP4	Sensitivity consistent with single-plex qPCR	[103]
GeXP platform	AIV-M, AIV-H1/H2/H3/H5/H6/H7/H9/H10-HA	100 copies/μL	[105]
GeXP platform	AIV-M, AIV-N1/N2/N3/N4/N5/N6/N7/N8/N9-NA	100 copies/reaction	[106]
GeXP platform	AIV-M, AIV-H5-HA, AIV-N1/N2/N6/N8-NA	100 copies/μL	[107]

Note: LOD values are presented as originally reported in the cited studies. Units differ across assays due to variable experimental conditions and are not directly comparable.

**Table 5:** ELISA Methods.

Method	Function	Target	Analytical LOD	Diagnostic Sensitivity	Diagnostic Specificity	Reference Method	Reference
Sandwich ELISA	Antigen detection	H9N2-HA protein	31.5 ng/mL for HA protein	93.2%	98.1%	RT-qPCR	[75]
Sandwich ELISA	Antigen detection	H9N2-HA protein	10^2.3^ TCID50 of H9N2 virus	98.9%	98.1%	Virus isolation	[114]
Sandwich ELISA	Antigen detection	AIV-H3 genotype-HA protein	Detectable down to a 1:10^5^ dilution of H3 virus stock (HA titer 2^8^)	100%	100%	Virus isolation, HI test, RT-PCR	[115]
Sandwich ELISA	Antigen detection	AIV-H6 genotype-HA protein	3.9 ng/mL for H6-HA protein and 0.5 HAU (HA units)/100 µL for live H6 subtype AIVs	NR	NR	NR	[116]
Fenobody-RANbody Sandwich ELISA	Antigen detection	NDV particles	10 ng of purified NDV particles	98.7%	NR	HA test	[72]
Digital ELISA	Antigen detection	AIV (H7N9) particles	7.8 fg/mL	NR	NR	NR	[12]
Epitope-Blocking ELISA	Antibody detection	Recombinant HA protein of AIV H5 genotypes	NR	98.0% (reference antisera)/99.1% (experimental antisera)	97.6% diagnostic specificity (100% analytical specificity)	HI test	[29]
cELISA	Antibody detection	Whole virus particle of AIV H7 genotypes	2^0^, 2^1^ and 2^−1^ HI titer for chicken, duck and peacock positive serum, respectively	100% (chicken), 98.10% (duck), 99.00% (peacock)	100% (chicken), 100% (duck), 100% (peacock)	HI test	[30]
pELISA	Antibody detection	Synthetic Polypeptide of IBV-S2 protein	NR	98.69%	80.0%	Commercial ELISA kit	[49]
pELISA	Antibody detection	Synthetic Polypeptide of IBV-S2 protein	NR	98.15 %	93.1%	Indirect immunofluorescence assay (IFA)	[68]
MAP-ELISA	Antibody detection	Synthetic Polypeptide of IBV-S protein	NR	94.85% (IBVM1), 92.65% (IBVM2); linear peptides: 90.44% (IBVP1), 88.24% (IBVP2)	92.05% (IBVM1), 90.91% (IBVM2); linear peptides: 88.64% (IBVP1), 85.23% (IBVP2)	Commercial ELISA kit	[117]
pELISA	Antibody detection	Synthetic Polypeptide of IBV-S protein	NR	98.30%	94.12%	IFA	[118]
Indirect ELISA	Antibody detection	Recombinant IBV-N protein	NR	90.16%	90.34%	Commercial ELISA test	[119]
Indirect ELISA	Antibody detection	Recombinant IBV-N Protein	NR	87%	100%	Commercial ELISA kit	[48]
Indirect ELISA	Antibody detection	Recombinant IBV-nsp5 Protein	NR	93.11% vs. IFA, 98.11% vs. IDEXX	95.38% vs. IFA, 95.00% vs. IDEXX	IFA and commercial ELISA kit (IDEXX)	[120]
Nanobody-cELISA	Antibody detection	IBV-N protein	Maximum dilution: 1:1000	98%	NR	Commercial ELISA kit	[47]
Nanobody-cELISA	Antibody detection	IBV-NP protein	NR	100%	98.6%	Positive samples	[121]
Blocking ELISA	Antibody detection	conserved structural protein	NR	100%	100%	HI test	[112]
Luminex xMAP	Antibody detection	Recombinant ILTV-gD and IBV-N protein	NR	84.44% (ILTV), 100% (IBV)	NR	ELISA	[59]

NR: Not reported.

**Table 6:** Performance comparison of diagnostic platforms for avian respiratory pathogens.

Diagnostic Platform	Target Type	Analytical Sensitivity	Multiplex Capacity	Total Turnaround Time	POCT Suitability	Recommended Application Scenarios
Single-plex/Multiplex qPCR	Nucleic acid	High	1–5 targets	1–2 h	Low	Routine surveillance/DIVA
dPCR	Nucleic acid	Ultra-high	1–4 targets	1–2 h	Low	Routine surveillance
RT-LAMP	Nucleic acid	High	1–2 targets	30–60 min	Moderate	Outbreak investigation
RT-RAA/RPA	Nucleic acid	High	1–2 targets	15–40 min	High	Outbreak investigation
CRISPR-Cas assay	Nucleic acid	Ultra-high	1–2 targets	30–60 min	High	Outbreak investigation
High-throughput chip/GeXP	Nucleic acid	Moderate	6–10 targets	4–6 h	Low	Routine surveillance
NGS/Nanopore sequencing	Nucleic acid	Untargeted (no fixed LOD)	Full pathogen coverage	1 h	Moderate	Routine surveillance/outbreak investigation/export certification
ELISA	Antigen/Antibody	Low	1–2 targets	2–4 h	Low	Routine surveillance/DIVA
ICS	Antigen	Low/semi-quantitative	Single target	10 min	High	Outbreak investigation
Lab-on-a-Chip	Nucleic acid	High	1–96 targets	10 min–5 h	Moderate	Routine surveillance/Outbreak investigation
Integrated Application of Smartphones	Nucleic acid/Antigen	High	3 targets	10–40 min	High	Outbreak investigation

Notes: Analytical sensitivity is a relative ranking based on typical LODs; values are not directly comparable across platforms due to varying units, with details in Table 1, Table 2, Table 3, Table 4 and Table 5. POCT suitability is a composite rating based on instrument requirements and operational simplicity.

## Data Availability

No new data were created or analyzed in this study. Data sharing is not applicable to this article.

## Abbreviations

The following abbreviations are used in this manuscript:

AIV	avian influenza virus
NDV	Newcastle disease virus
IBV	infectious bronchitis virus
ILTV	infectious laryngotracheitis virus
POCT	point-of-care diagnostic techniques
LAMP	loop-mediated isothermal amplification
HA	Hemagglutinin
NA	Neuraminidase
M	Matrix
NP	Nucleoprotein
RAA	recombinase-aided amplification
RPA	recombinase polymerase amplification
ELISA	enzyme-linked immunosorbent assay
HI	Hemagglutination inhibition
P	phosphoprotein
F	fusion protein
HN	hemagglutinin-neuraminidase protein
S	spike protein
IBV-M	membrane protein
E	envelope protein
N	nucleocapsid protein
HVR	hypervariable region
TCoV	turkey coronavirus
RdRp	RNA-dependent RNA polymerase
GaHV-1	Gallid herpesvirus 1
TK	thymidine kinase
vCKBP	viral chemokine-binding protein
WOAH	World Organisation for Animal Health
SPF	specific-pathogen-free
VN	virus neutralization
DIVA	differentiate infected from vaccinated animals
RT-PCR	reverse transcription PCR
DTMUV	duck Tembusu virus
aMPV	avian metapneumovirus
FWPV	fowlpox virus
REV	reticuloendotheliosis virus
GPV	goose parvovirus
GoAstV	goose astrovirus
HRM	high-resolution melting
dPCR	digital PCR
ddPCR	droplet digital PCR
RT-LAMP	reverse transcription LAMP
LFD	lateral flow dipstick
AuNP	gold nanoparticle
NALF	nucleic acid lateral flow immunochromatographic test strips
UDG	uracil-DNA glycosylase
NGS	next-generation sequencing
mNGS	Meta-genomic NGS
ConA	concanavalin A
pELISA	peptide-based enzyme-linked immunosorbent assay
MAP	multi-antigen peptide
eGFP-NT	eGFP reporter virus
HRP	horseradish peroxidase
cELISAs	competitive enzyme-linked immunosorbent assays
LOC	lab-on-a-chip
AI	artificial intelligence
SNP	single-nucleotide polymorphism
